# Plant Distribution Data Show Broader Climatic Limits than Expert-Based Climatic Tolerance Estimates

**DOI:** 10.1371/journal.pone.0166407

**Published:** 2016-11-21

**Authors:** Caroline A. Curtis, Bethany A. Bradley

**Affiliations:** 1 Graduate Program in Organismic and Evolutionary Biology, University of Massachusetts, Amherst, MA, 01003, United States of America; 2 Department of Environmental Conservation, University of Massachusetts, Amherst, MA, 01003, United States of America; Universidad de la Republica Uruguay, URUGUAY

## Abstract

**Background:**

Although increasingly sophisticated environmental measures are being applied to species distributions models, the focus remains on using climatic data to provide estimates of habitat suitability. Climatic tolerance estimates based on expert knowledge are available for a wide range of plants via the USDA PLANTS database. We aim to test how climatic tolerance inferred from plant distribution records relates to tolerance estimated by experts. Further, we use this information to identify circumstances when species distributions are more likely to approximate climatic tolerance.

**Methods:**

We compiled expert knowledge estimates of minimum and maximum precipitation and minimum temperature tolerance for over 1800 conservation plant species from the ‘plant characteristics’ information in the USDA PLANTS database. We derived climatic tolerance from distribution data downloaded from the Global Biodiversity and Information Facility (GBIF) and corresponding climate from WorldClim. We compared expert-derived climatic tolerance to empirical estimates to find the difference between their inferred climate niches (ΔCN), and tested whether ΔCN was influenced by growth form or range size.

**Results:**

Climate niches calculated from distribution data were significantly broader than expert-based tolerance estimates (Mann-Whitney p values << 0.001). The average plant could tolerate 24 mm lower minimum precipitation, 14 mm higher maximum precipitation, and 7° C lower minimum temperatures based on distribution data relative to expert-based tolerance estimates. Species with larger ranges had greater ΔCN for minimum precipitation and minimum temperature. For maximum precipitation and minimum temperature, forbs and grasses tended to have larger ΔCN while grasses and trees had larger ΔCN for minimum precipitation.

**Conclusion:**

Our results show that distribution data are consistently broader than USDA PLANTS experts’ knowledge and likely provide more robust estimates of climatic tolerance, especially for widespread forbs and grasses. These findings suggest that widely available expert-based climatic tolerance estimates underrepresent species’ fundamental niche and likely fail to capture the realized niche.

## Introduction

Understanding the factors that define species niches has long been a central theme in ecology, beginning with Joseph Grinnell’s initial description of the niche as an ecological space sufficient for the survival of a single species [1]. Interest in the ecological niche was further developed by G. E. Hutchinson, who refined the niche concept by separating the fundamental niche (the multidimensional environmental conditions in which a population could exist) from the realized niche (the biotic and abiotic conditions in which a species actually does exist) [2]. In the decades following Hutchinson’s statements, there began the development of research focused on modeling species distributions and disentangling the factors that define the fundamental and realized niches (e.g. [3,4]). Recently, more sophisticated techniques have been applied to defining a species’ niche [5] and species distribution models (SDMs) have become increasingly useful tools for identifying a species’ habitat, projecting distribution changes in response to climate [6], and mapping habitat areas of importance for biodiversity conservation [7,8] and those at risk from environmental threats [9].

However, SDMs parameterized from species distributions are likely to underestimate climatic tolerance because species are not in climatic equilibrium (i.e., they are not present in all climatically suitable locations [2] and/or distribution data are unevenly collected and reported, thereby underrepresenting the total distribution [10]. Some of the earliest works on SDMs highlighted the need to consider species occurrences outside the natural range [11]. In doing so, SDMs will more closely approximate the fundamental niche, allowing researchers to draw more accurate conclusions about the potential for range shifts in response to climate change. Underestimating the climatic niche, which may be the result of parameterizing models with a subset of a species’ range, causes models to miss suitable climatic space under current and future conditions, potentially exaggerating habitat loss and associated risk to species.

Several lines of evidence suggest that species distributions underestimate climatic tolerance. For example, studies comparing niche space in the native and non-native ranges have often shown that non-native occurrences expand the climatic niche (i.e. show a lack of niche conservatism) [12–15]. Other studies have found that regional distributions are heavily influenced by factors other than climate including dispersal barriers [16–19], introduction history [20] and biotic interactions [16,21,22]. Collectively, these influences on species realized niches cause distributions to underestimate climatic tolerance. Therefore, correlative models, which typically rely on climatic tolerance derived from species distributions, might be of limited utility for projecting suitable habitat in future or novel environmental conditions because they lack the causal information that can be derived from functional traits included in mechanistic models [23].

One potential solution is to avoid the problems inherent in species distribution data by building SDMs based on alternative climatic tolerance data such as experimentally gathered physiological tolerance data [24–27] or expert-based estimates of growth requirements. Spatial models based on physiological tolerance information for a species are more likely to identify the fundamental niche, or all conditions where a species can survive, rather than the smaller realized niche based on where the species currently exists geographically. In one example, Buckley et al. [28] parameterized spatial models for the eastern fence lizard (*Sceloporus undulatus*) in the USA based on empirically measured foraging energetics, biophysical thresholds, and demography along with downscaled climate data to project suitable habitat under current and future climate conditions. In another example, critical maximum thermal thresholds were measured experimentally for forest-dwelling ant species and used to model response to warming across a latitudinal gradient [29]. The mechanistic approach has also been used to model the dispersal and population growth potential for the invasive cane toad in Australia [30].

However, measuring physiological tolerance limits requires time intensive field- and/or lab-based sampling and, as a result, those data are only available for a few well-studied taxa. Similarly, expert-opinion data are typically limited to crops, ornamental species or those used in conservation and restoration. The USDA PLANTS database provides standardized information primarily designed to support land conservation activities and consists of estimates of climatic tolerance for approximately 2500 plant species and cultivars in its ‘characteristics’ data. These data are based on expert knowledge rather than experimental manipulations and are, therefore, considered estimates of the range of conditions under which the species can survive. However, given that this database is among the first large-scale compilation of freely available, easily accessible plant climatic tolerance estimates, it provides an appealing alternative to species distribution data. The USDA PLANTS database is also a primary source for some plant traits, including temperature tolerance and precipitation requirement, archived in the TRY database (https://www.try-db.org; [31]). TRY is an important repository for plant trait data which includes over 1,000 traits and 100,000 plant species [31], and reported estimates of climatic tolerance could easily be interpreted as ‘fundamental’ growth requirements. Moreover, climatic tolerance estimates from USDA PLANTS are used to select species for conservation and restoration of ecosystems across the US. Thus, it is important to understand how well these expert-derived climatic tolerances perform in terms of capturing suitable climatic limits relative to species’ distributions.

Here, we compare climatic niches inferred from two commonly used data sets: the USDA PLANTS database, and herbarium records from the Global Biodiversity Information Facility (GBIF). Observations available on GBIF represent both species’ native distribution as well as records outside of the native range (e.g., accidental movement or purposeful plantings of species). Therefore, these data provide useful insight about species’ climatic tolerance. For example, the climatic niche of commercial Eucalypt species was better approximated by integrating global GBIF records with native range data from the Atlas of Living Australia (ALA) [32] and the extinction risk for over 7000 woody plant species was modeled from compiled GBIF, Forest Inventory and Analysis (FIA) and permanent sampling plot data [33]. We calculated a comparative niche value (hereafter ΔCN) for each species as the difference between climatic niche defined by expert-based climatic tolerance estimates and those derived from climate conditions associated with distribution data. First, we ask how climatic niches calculated from expert-based climatic tolerance estimates compare to those calculated from distribution data. Second, we ask whether range size or growth form influence ΔCN and lastly, we ask whether species growth form influences ΔCN.

## Methods

### Expert-based climatic tolerance data

The USDA PLANTS database provides detailed information about the traits and growth requirements of approximately 2,500 vascular plants in their ‘characteristics’ data. Included in these characteristics data are estimates of species’ tolerance to absolute (i.e. record) minimum temperature (for perennial species and annual species with dominant growing seasons in fall, winter, and spring), and minimum and maximum precipitation tolerance (for all species). Climatic tolerance is estimated based on expert knowledge of historical and current species ranges within the USA and associated historical climate conditions. We downloaded the characteristics data, including growth form, for all species for which minimum temperature, and/or minimum and maximum precipitation were available and refer to these data hereafter as climatic tolerance estimates (S1 and S2 Tables).

### Herbarium records

We searched the Global Biodiversity Information Facility (GBIF) for all species with climatic tolerance estimates using the rgbif package for R [34] and downloaded all georeferenced records. Because the climatic tolerance estimates are based on expert knowledge of tolerance within the USA, we created a USA distribution dataset by restricting the records for each species to the coterminous USA, Hawaii, and Alaska (hereafter, USA herbarium). We excluded species with five or fewer records from the analyses. Taxonomic discrepancies between the USDA PLANTS database and the GBIF records were resolved using the Integrated Taxonomic Information System (ITIS, http://www.itis.gov). In cases where a single species was reported by two names (i.e., one name was identified as a synonym of the other using ITIS), the taxonomy given by the USDA PLANTS database was retained. Data representing current climate (1950–2000) were obtained from WorldClim (http://worldclim.org) as interpolated climate surface layers at 10 arc-minute (approximately 344 km^2^ at the equator) spatial resolution [35] and extracted to herbarium records. WorldClim data were used to encompass both the coterminous USA as well as Hawaii and Alaska. For each species, we used the climate data associated with occurrence records to calculate the 95^th^ percentile of minimum temperature and minimum and maximum precipitation (S1 and S2 Tables). We present the 95^th^ percentile to avoid biasing our calculations due to inaccurate outliers in the distribution dataset, although we also calculated results associated with absolute minimum and maximum values (S1 Fig). We calculated range size based on the area within a convex hull surrounding the occurrence records for each species.

### Climate corrections and comparisons

The climatic tolerance estimates are based on extreme values of each climate variable (e.g., absolute minimum temperature) while the WorldClim dataset is based on temporal averages from 1950–2000. In order to make the datasets comparable, we transformed the average values into extreme values based on linear corrections using climate time series available for the US.

Minimum precipitation climatic tolerance estimates are reported as the cumulative annual precipitation that occurs 20% of the time at the driest weather station (i.e. the annual precipitation value corresponding to the 20^th^ percentile over a multi-year period). In the WorldClim dataset, precipitation is recorded as cumulative monthly precipitation averaged over the time period of 1950–2000. We adjusted the WorldClim results to make them more directly comparable to the climatic tolerance estimates of minimum precipitation by calculating climate transformations based on time series of PRISM climate interpolations [36]. PRISM data are available as time series of monthly precipitation from 1981–2013 for the continental US. We used these time series to calculate the 20^th^ percentile of annual precipitation and compared these data to the PRISM average annual precipitation using 50,000 random points in the continental US to calculate and apply linear gain and offset corrections.

Maximum precipitation climatic tolerance estimates are reported as the mean annual precipitation at the wettest weather station in the species’ range as defined by expert knowledge. WorldClim monthly maximum temperature is calculated as the temporally averaged (1950–2000) mean monthly temperature plus half of the monthly temperature range [35]. As the two measures are both based on average precipitation, we considered them comparable and did not apply a climate transformation to the WorldClim maximum precipitation results.

Minimum temperature climatic tolerance estimates are reported as either the lowest recorded temperature from the historical range or the lowest January temperature recorded from weather stations within the current range. WorldClim monthly minimum temperature is calculated as the mean monthly temperature minus half of the monthly temperature range [35]. These data are then temporally averaged (1950–2000) to estimate minimum temperature. To adjust the WorldClim results for minimum temperature and make them comparable to the climatic tolerance estimates, we calculated a climate transformation based on time series of weather station climate records [37]. We compiled a time series (1950–2000) of daily January minimum temperature from over 80 weather stations throughout the USA (S2 Fig, S3 Table). From daily temperature data, we calculated the absolute lowest January minimum temperature and the 1950–2000 average January minimum temperature from which the linear gain and offset corrections were calculated and applied to the WorldClim data. We capped the minimum temperature at -60° C, which approximates the coldest temperatures measured in the USA.

## Analyses

We used Mann-Whitney-Wilcoxon tests to compare the climate niches derived from the climatic tolerance estimates and the herbarium records. We calculated ΔCN for each species and each of the three climate variables (maximum precipitation, corrected minimum precipitation, and corrected minimum temperature). For consistent visual comparison, ΔCN was calculated for all climate variables such that positive values indicate a broader climatic niche measured from the herbarium data. We used linear regressions to test for a relationship between species range size and the magnitude of ΔCN for each climate variable. We used Kruskal-Wallis tests and post-hoc Kruskal Nemenyi tests to determine if ΔCN varied by growth form.

## Results

From the USDA PLANTS database, minimum and maximum precipitation data were available for 2053 species and minimum temperature data were available for 2080 species (excluding summer annuals). Forbs/herbs and trees were the most common growth form in the data comprising 28% and 27% of the species, respectively. Grasses (23%) and shrubs (19%) were also well represented while vines represented only 3% of the species in the dataset. Of those species with climatic tolerance estimates for minimum and maximum precipitation, and minimum temperature, six or more GBIF herbarium records within the USA were available for 1860 and 1870 species, respectively.

**Fig 1:**
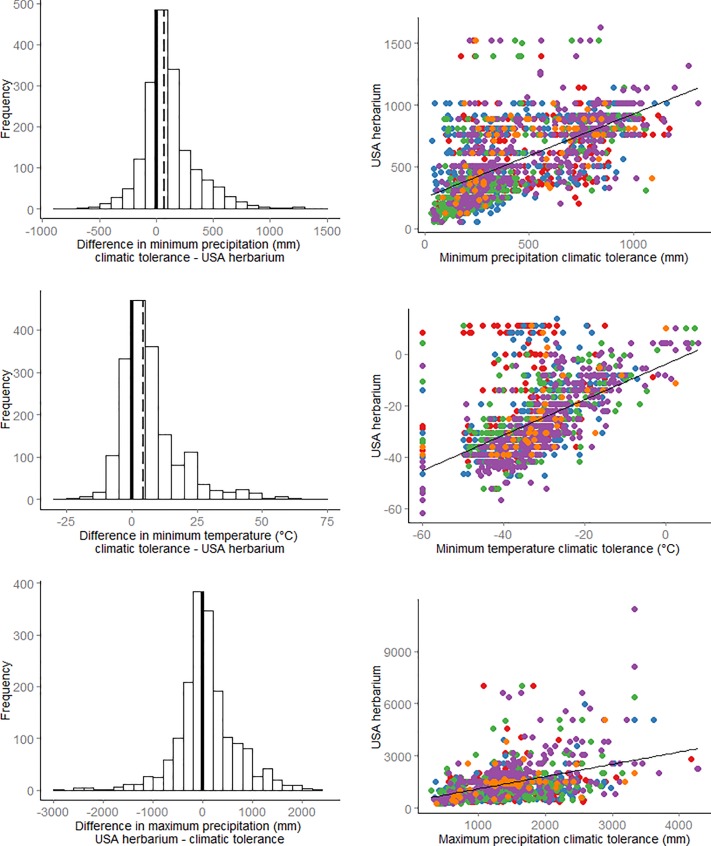
Frequency distributions of comparative niche values (ΔCN). For all climate variables, herbarium records from GBIF tend to estimate broader climatic niches than climatic tolerance estimates. (A-C) Histogram values to the right of zero (solid line) indicate species with broader climatic niches from herbarium records. The dashed line indicates the median ΔCN. (D-F) Scatterplots show the raw values of climatic tolerance derived from expert-estimates vs. herbarium records. Colored points differentiate species’ primary growth forms (red = Forb, blue = Grass, green = Shrub, purple = Tree, orange = Vine). Points below the 1:1 line for minimum precipitation (D) and minimum temperature (E) and above the 1:1 line for maximum precipitation (F) have broader niches described by herbarium records.

Mann-Whitney-Wilcoxon tests showed that the distribution of climatic niches measured from the climatic tolerance estimates and herbarium records are significantly different for minimum precipitation (W = 1991976, p-value << 0.001), minimum temperature (W = 1993790, p-value << 0.001), and maximum precipitation (W = 1523031, p-value = 0.04) (Fig 1).

For all climate variables, we measured a broader climate niche from the herbarium records than from the climatic tolerance estimates. Minimum and maximum precipitation values derived from herbarium records were broader (i.e. lower minimum and higher maximum) than climatic tolerance estimates for 71% and 52% of species, respectively (Fig 1A and 1B). The mean minimum and maximum precipitation values from the herbarium records were 110 mm (median: 72 mm) lower and 24 mm (median: 13 mm) higher than climatic tolerance estimates, respectively. Similarly, 74% of species had lower minimum temperature recorded from the herbarium records than from the climatic tolerance data (Fig 1C), with a mean difference of 7° C (median 4.5° C). For only one growth form was this general trend not supported. ΔCN values for maximum precipitation were generally negative for trees, with the climate niche inferred from climatic tolerance estimate larger by an average of 300 mm. When all USA distribution data were considered (not just the 95^th^ percentile), the pattern was even more pronounced, with the vast majority of species showing a broader climate niche from herbarium records (S1 Fig).

**Fig 2:**
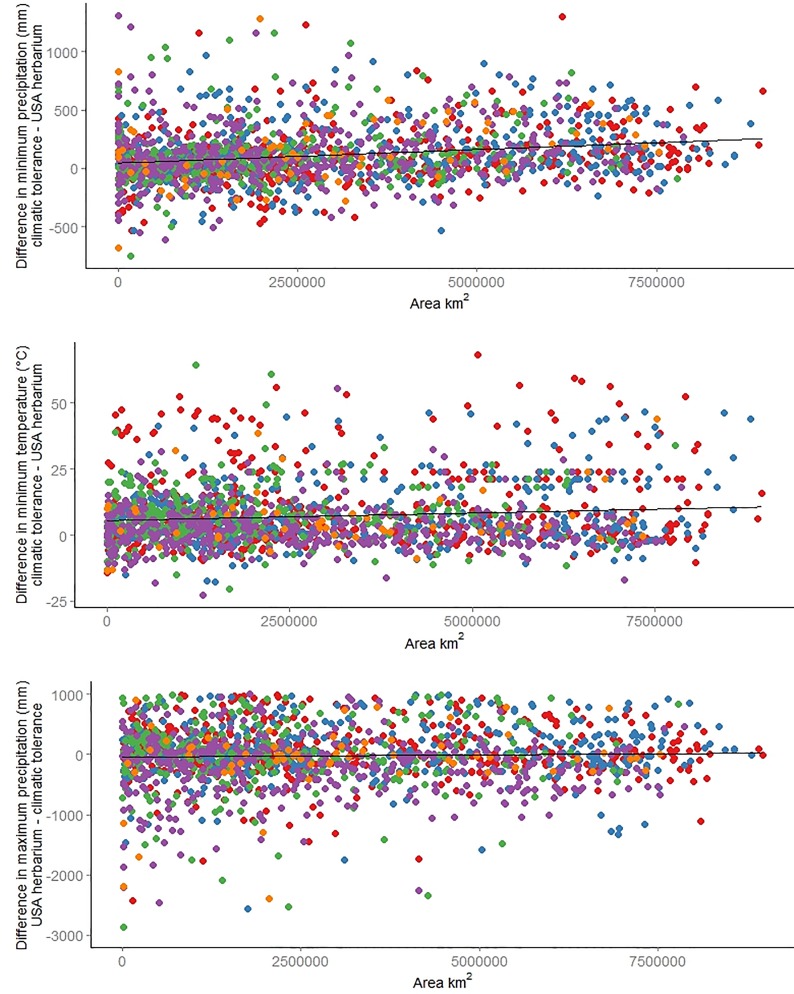
Change to ΔCN relative to species range size. Herbarium records estimate an increasingly broader climatic tolerance relative to expert-based climate tolerance (ΔCN) as range size increases. A significant positive relationship was found for minimum precipitation (A) and minimum temperature (B). The trend was similar, although not significant, for maximum precipitation (C). ΔCN values for maximum precipitation were truncated at -3000 for ease of interpretation. Colored points differentiate species’ primary growth forms (red = Forb, blue = Grass, green = Shrub, purple = Tree, orange = Vine).

Species range size was significantly positively related to ΔCN for minimum precipitation (df = 1779, p-value << 0.001; Fig 2A) and minimum temperature (df = 1783, p-value << 0.001; Fig 2B). In other words, for species with larger ranges, there was a greater difference between the two datasets (with herbarium records consistently broader) than for species with smaller ranges. Although there was a slight positive trend between range size and ΔCN for maximum precipitation, the trend was not significant (df = 1779, p-value = 0.606, Fig 2C).

**Fig 3:**
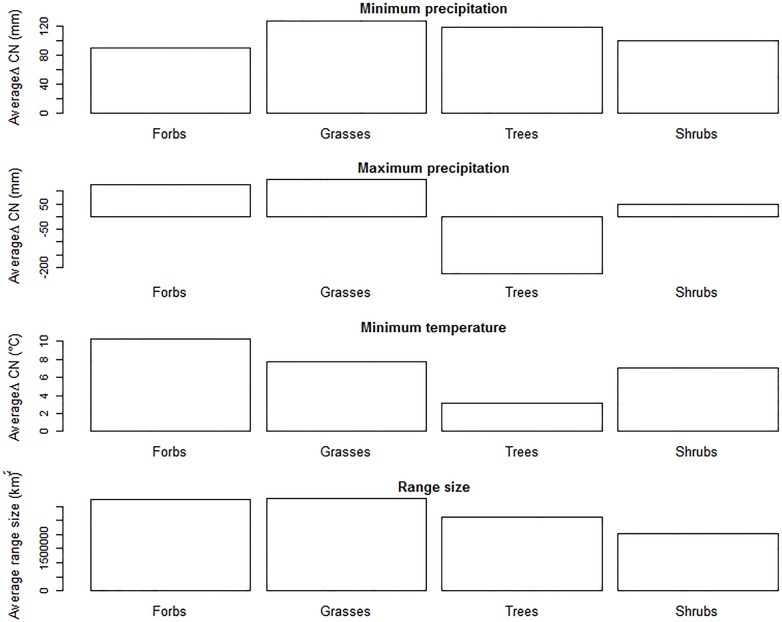
Difference between calculated climate niche (ΔCN) varies by growth form. ΔCN was primarily positive (distribution data suggested a broader tolerance than expert-based climate tolerance), but differed by growth form for all climate variables, suggesting that the effectiveness of distribution data for identifying the climatic niche might vary with growth form. Plant growth forms had significantly different average ΔCN values for (A) minimum precipitation, (B) maximum precipitation and (C) minimum temperature. Average range size also differed by growth form for all data (D).

We found that ΔCN differed by growth form for minimum precipitation (KW X^2^ = 79, df = 4, p << 0.001), maximum precipitation (KW X^2^ = 35, df = 4, p << 0.001), and minimum temperature (KW X^2^ = 133, df = 4, p << 0.001) (Fig 3A–3C). Because of the small number of vine species, we excluded vines from the growth form analyses. Grasses and/or forbs tended to have the broadest range size as well as the largest ΔCN values, with herbarium records consistently broader than climatic tolerance estimates. In contrast, trees and shrubs tended to have narrower ranges and smaller (although still positive) ΔCN values. In only one case was this general trend not supported. ΔCN values for maximum precipitation were negative for trees, with the climate niche inferred from climatic tolerance estimates larger by an average of 300 mm. We also found that range size differed by growth form (KW X^2^ = 79, df = 4, p-value << 0.001) (Fig 3D).

## Discussion

Understanding the extent to which commonly used data sources approximate species’ fundamental niche is an important step toward creating realistic predictions of suitable habitat. Distribution data are likely to underestimate the fundamental niche because species ranges are not in climatic equilibrium [2]. Distributions are limited not only by climate conditions, but also by dispersal barriers, introduction history and biotic interactions (e.g. [15,18,20,21]). These concerns have prompted proposals to use alternative estimates of climatic tolerance in lieu of distribution data when parameterizing spatial models [24,25,38], assuming that expert knowledge or lab-based measurements will provide a broader approximation of climatic tolerance. However, our results show that distribution data in the US describe a consistently broader climatic niche than climatic tolerance estimates available for over 1800 plants (Fig 1).

Distribution data suggest a broader climatic tolerance for all three of the climate variables tested and the magnitude of the difference was substantial. Distribution data suggest that the average plant can withstand an extreme minimum temperature 7° C lower than estimated by experts. These records also infer lower drought tolerance (20^th^ percentile of annual precipitation) of 24 mm. Moreover, these results are based on a conservative approach of using the 95^th^ percentile of minimum and maximum precipitation and minimum temperature for each species. The results are even more pronounced (15° C lower minimum temperature, 250 mm lower drought tolerance) if we use a less conservative approach and include all distribution data in the analyses (S1 Fig).

The one exception to this trend is ΔCN measured for maximum precipitation among tree species. One possible explanation for this inconsistent result is that, for a few species with distributions in areas of high precipitation, an extreme value of maximum precipitation was reported in the USDA PLANTS characteristics. For example, white mangrove (*Laguncularia racemosa*) has a maximum precipitation ΔCN value of -1082 (expert estimate: 2337 mm; distribution data: 1255 mm). It is possible that experts over-estimate maximum precipitation for species located near strong precipitation gradients. For most species, our results suggest that plant distribution data are likely to produce a comparable or broader estimate of climatic habitat than currently available climatic tolerance estimates.

Although we find that herbarium records produce broader estimates of climatic tolerance, this does not necessarily suggest that distribution data are doing a good job of approximating the climate niche for most species. Instead, the differences between the two datasets may result from poor climatic estimates in both datasets. Currently, there are few repositories for climatic tolerance data and limited information available due to the difficulty of experimentally deriving tolerance across many species, particularly for some long-lived plants. Climate conditions experienced by species is also likely to be influenced by local topography and land cover [39], which cannot be effectively captured at coarser spatial resolutions such as the one used in this analysis. Moreover, climatic tolerances vary across species distributions as a result of local adaptation and provenance variations, making it all the more challenging to identify tolerance limits [40]. Our findings suggest that the USDA PLANTS characteristics data underestimate climatic tolerance for the majority of species. Where these data are associated with the TRY database [31], the same result is likely to be true. Alternatively, discrepancies between the data sets might be due to the biases inherent in their collection. Herbarium records tend to reflect species range edges, be skewed toward populations of rare species, and be biased toward populations that are convenient to sample (e.g., close to trails or roads). Conversely, because the expert-based climatic tolerance estimates are used for conservation and restoration planning, they might be representative of the average climate conditions in areas where the plant has the highest likelihood of thriving. These findings are important both for biogeographers interested in the niche as well as land managers involved in conservation and restoration projects, which rely on climatic tolerance data (see Brown et al. 2008 for examples). In order to produce more robust estimates of climatic tolerance, and to better infer how well distribution data approximate fundamental tolerance limits, more physiological data based on experimental manipulations are needed.

Species with larger range sizes had increasingly broader climatic tolerance estimates derived from distribution data (Fig 2). The linear regression models showed that for all climate variables, there was a positive relationship between range size and ΔCN values, although this relationship was not significant for maximum precipitation. This increasing disparity between the two datasets suggests that climatic tolerance inferred from species with small ranges is likely to underestimate the climatic niche more severely than widespread species. This result may be partially due to errors associated with modeling species with few occurrence points [41]. For species with a small number of occurrence records, SDMs tend to produce locally accurate models of suitable climate space but perform poorly at projecting outside the range of sampled conditions [42,43]. Several recent studies have also highlighted the importance of range size when considering how well distribution data approximate climatic tolerance. For example, species with larger ranges were less likely to show a climatic niche expansion when introduced outside their native ranges [14,15,19]. Similarly, species with larger ranges were more likely to fill in available habitat at range margins [44]. Our results support these findings, suggesting that distribution data are more apt to produce robust estimates of climatic tolerance when species have large ranges.

Our comparison of ΔCN values between plant growth forms suggests that distribution data for different taxonomic groups have unequal climatic niche filling (Fig 3A–3C). Previous studies suggest that niche filling might differ between groups. Araújo et al. [45] found a higher degree of niche filling among European vascular plants relative to reptile and amphibian species. In contrast, the mean range filling was found to be less than 40% for 55 native European tree species indicating that distributions were likely heavily influenced by non-climatic factors such as dispersal constraints [17]. Our results suggest that distribution data for forbs and grasses generally encompass a broader climatic niche than distribution data for shrubs and trees.

However, a broader climatic niche for widespread forbs and grasses may not be generalizable across all plants. Shorter generation times might enable faster dispersal and greater niche filling amongst grasses and forbs relative to shrubs and trees. The species included in the USDA PLANTS database are planted throughout the USA for specific conservation or restoration needs. For example, the database is used in support of the Natural Resources Conservation Service (NRCS) Plant Materials Program, which selects conservation plant species and implements planting protocols for ecoregions in the USA. As a result, the broader climatic niches associated with forbs and grasses may be partially due to human introduction rather than to natural dispersal ability. Differences between the datasets could also occur if experts report an approximation of the average climate space (i.e., the climate space in which a species will thrive in conservation/restoration projects), while herbarium records are more likely to include rare specimens which could lead to exaggerated climate niches relative to the core distribution.

For narrow range species in particular, combining distribution data with climatic tolerance information will likely improve model projections. However, we caution the biogeography community that climatic tolerance estimates available through USDA PLANTS or TRY [31] should not necessarily be interpreted as ‘truth’. The expert-based estimates evaluated here appear overly conservative, but even experimentally derived tolerance is influenced by variation within populations [46] and often differs across species ranges [40,47]. The combination of both distribution and climatic tolerance estimates is more likely than strictly correlative models to approximate a species’ fundamental niche, thereby improving projections of suitable habitat under novel combinations of climatic conditions [48]. A combined approach also allows the modeler to identify areas of higher confidence within the projection (e.g., where the models overlap) and areas where the predictor variables failed to capture the factors limiting the species’ distribution (e.g., where models differ [49,50]). For example, species-specific temperature tolerance data were combined with distribution data to model macroalgae survival [51] and the geographic responses of UK butterflies to climate change [52]. Similarly, variation in climatic factors generalized to a single widespread tree species were used to parameterize a hybrid model for six tree species in the Pacific Northwest USA [53].

Spatial models are powerful tools for understanding how species are likely to respond to global change, and independent climatic tolerance data are increasingly used to improve estimates derived from distribution data. Biogeographers recognize the need to develop more integrated, mechanistic models but the cost of developing mechanistic models precludes their use for large numbers of species. For the majority of the plants we evaluated, distribution data suggest a broader climatic tolerance than expert-based climate tolerance estimates. For widespread species in particular, distribution data produce a better approximation of climatic tolerance.

## Supporting Information

S1 Fig ΔCN calculated for entire dataset (rather than the conservative 95% threshold) reveal broader climatic tolerance estimated from herbarium records for all climate variables. Frequency distributions of the comparative niche values (ΔCN) calculated for the entire dataset (rather than the 95^th^ percentile) show that herbarium records tend to estimate broader climatic niches than physiological tolerance estimate. Positive differences indicate broader climatic niches measured from the herbarium records. The solid line indicates zero and the dashed lines indicate the median ΔCN. Herbarium records predicted a lower minimum temperature tolerance (A) for 92% of species (median ΔCN = 12.5°C). Lower minimum (B) and maximum precipitation (C) was found for 91% and 78% of species, respectively (median ΔCN = 209 mm and 357 mm, respectively).
(TIF)

S2 Fig Locations of weather stations used to calculate the linear gain and offset between the Worldclim and PRISM datasets. Weather stations were selected from each state in the USA, with additional stations selected as needed to fill gaps in the climate space.
(TIF)

S1 Table Estimates of climatic tolerance, and calculations of ΔCN and range size for all plant species for which minimum temperature data were available. (CSV)

S2 Table Estimates of climatic tolerance, and calculations of ΔCN and range size for all plant species for which minimum and maximum precipitation data were available. (CSV)

S3 Table Weather station name and locality data. (DOCX)
