# Supplementary figures and images for: Plant Distribution Data Show Broader Climatic Limits than Expert-Based Climatic Tolerance Estimates
Source: PLoS One. 2016 Nov 21;11(11):e0166407. doi: 10.1371/journal.pone.0166407 (PMC5117642; doi:10.1371/journal.pone.0166407)

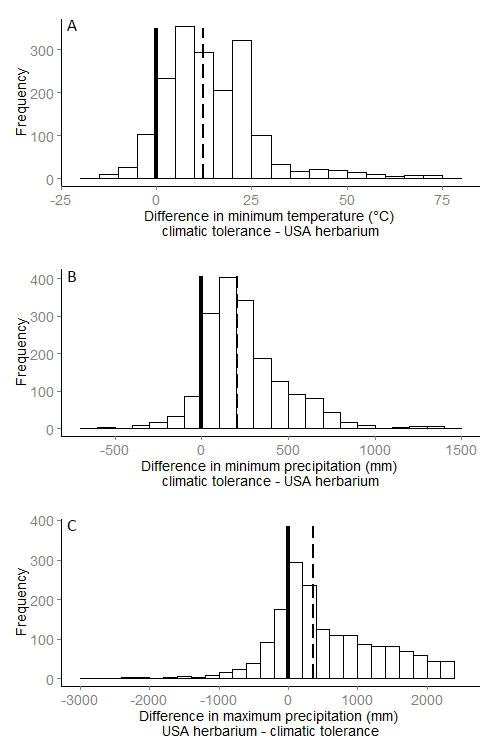

Supplement: S1 Fig — Frequency distributions of the comparative niche values (ΔCN) calculated for the entire dataset (rather than the 95th percentile) show that herbarium records tend to estimate broader climatic niches than physiological tolerance estimate. Positive differences indicate broader climatic niches measured from the herbarium records. The solid line indicates zero and the dashed lines indicate the median ΔCN. Herbarium records predicted a lower minimum temperature tolerance (A) for 92% of species (median ΔCN = 12.5°C). Lower minimum (B) and maximum precipitation (C) was found for 91% and 78% of species, respectively (median ΔCN = 209 mm and 357 mm, respectively). (TIF) [file pone.0166407.s001.tif]

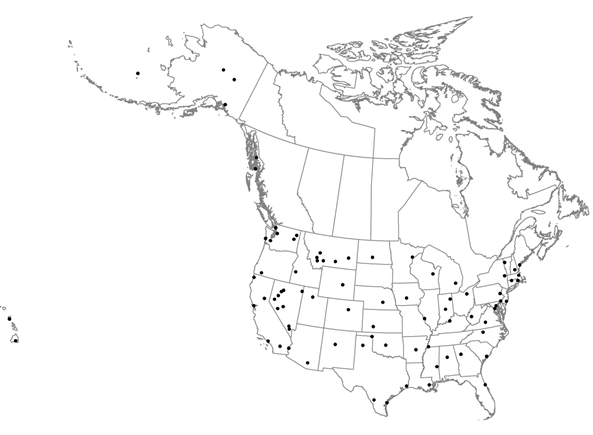

Supplement: S2 Fig — Weather stations were selected from each state in the USA, with additional stations selected as needed to fill gaps in the climate space. (TIF) [file pone.0166407.s002.tif]
