# Supplementary material for: Plant Distribution Data Show Broader Climatic Limits than Expert-Based Climatic Tolerance Estimates
Source: PLoS One. 2016 Nov 21;11(11):e0166407. doi: 10.1371/journal.pone.0166407 (PMC5117642; doi:10.1371/journal.pone.0166407)
Supplement: S3 Table — (DOCX) [file pone.0166407.s005.docx]

**S5 Table : Weather station name and locality data.**

| Station Name | State | LATITUDE | LONGITUDE |
| --- | --- | --- | --- |
| Albany Airport | NY | 42.75 | -73.8 |
| Albuquerque International Airport | NM | 35.05 | -106.617 |
| Allentown Lehigh Valley International Airport | PA | 40.65 | -75.4333 |
| Amarillo International Airport | TX | 35.23333 | -101.7 |
| Anacortes | WA | 48.51667 | -122.617 |
| Annette Weather Service Office Airport | AK | 55.03333 | -131.567 |
| Atlanta Hartsfield International Airport | GA | 33.65 | -84.4167 |
| Atlantic City International Airport | NJ | 39.45 | -74.5833 |
| Baltimore Washington International Airport | MD | 39.18333 | -76.6667 |
| Billings International Airport | MT | 45.8 | -108.533 |
| Birmingham Airport | AL | 33.45 | -86.85 |
| Bismarck Municipal Airport | ND | 46.7825 | -100.757 |
| Blythe Airport | CA | 33.61667 | -114.6 |
| Boise Air Terminal | ID | 43.56667 | -116.241 |
| Boston Logan International Airport | MA | 42.36667 | -71.0167 |
| Bozeman Montana SU | MT | 45.67056 | -111.05 |
| Burlington International Airport | VT | 44.46667 | -73.15 |
| Butte Bert Mooney Airport | MT | 45.95 | -112.5 |
| Casper Natrona CO International Airport | WY | 42.8975 | -106.464 |
| Centralia | WA | 46.71667 | -122.95 |
| Charleston International Airport | SC | 32.9 | -80.0333 |
| Charleston Yeager Airport | WV | 38.36667 | -81.6 |
| Chewelah | WA | 48.25 | -117.717 |
| Cleveland Hopkins International Airport | OH | 41.4 | -81.85 |
| Concord Municipal Airport | NH | 43.2 | -71.5 |
| Corpus Christi NAS | TX | 27.7 | -97.2667 |
| Davenport | WA | 47.65 | -118.15 |
| Daytona Beach International Airport | FL | 29.18333 | -81.05 |
| Denver Stapleton | CO | 39.75 | -104.867 |
| Des Moines International Airport | IA | 41.53333 | -93.65 |
| Desert National WL Range | NV | 36.43778 | -115.36 |
| Desert Resorts Regional Airport | CA | 33.63333 | -116.167 |
| Dodge City Regional Airport | KS | 37.76667 | -99.9667 |
| Duluth International Airport | MN | 46.83694 | -92.21 |
| Encinal | TX | 28.05 | -99.35 |
| Fairbanks International Airport | AK | 64.83333 | -147.717 |
| Flint Bishop International Airport | MI | 42.96667 | -83.75 |
| Follett | TX | 36.43333 | -100.133 |
| Fort Wayne International Airport | IN | 41 | -85.2 |
| Grand Island Central NE Regional Airport | NE | 40.96667 | -98.3167 |
| Grayland | WA | 46.8 | -124.083 |
| Green Bay Austin Straubel International Airport | WI | 44.487 | -88.138 |
| Greensboro Piedmont Triad International Airport | NC | 36.08333 | -79.95 |
| Hartford Bradley International Airprot | CT | 41.93806 | -72.6825 |
| Hawaii Volcano National Park HQ 54 | HI | 19.43306 | -155.26 |
| Helena Regional Airport | MT | 46.6 | -112 |
| Honolulu International Airport | HI | 21.33333 | -157.917 |
| Imlay | NV | 40.66667 | -118.15 |
| Indianapolis International Airport | IN | 39.73333 | -86.2667 |
| Kentfield | CA | 37.95 | -122.55 |
| Lower Klamath | CA | 41.52167 | -124.032 |
| Lahontan Dam | NV | 39.46667 | -119.067 |
| Las Vegas McCarran International Airport | NV | 36.08333 | -115.167 |
| Little Roack Airport Adams Field | AR | 34.73333 | -92.2333 |
| Los Angeles International Airport | CA | 33.93333 | -118.383 |
| Louisville International Airport | KY | 38.18333 | -85.7333 |
| Lovelock Derby Field | NV | 40.06667 | -118.55 |
| Lynchburg Regional Airport | VA | 37.33333 | -79.2 |
| Makaweli 965 | HI | 21.91667 | -159.633 |
| Medford Gogue Valley International Airport | OR | 42.36667 | -122.867 |
| Memphis International Airport | TN | 35.05 | -89.9833 |
| Meridian Key Field | MS | 32.33333 | -88.75 |
| Miles City 1.2 ENE | MT | 46.43333 | -105.867 |
| Mina | NV | 38.38333 | -118.1 |
| Monroe | WA | 47.85 | -121.983 |
| Montello | NV | 41.26667 | -114.2 |
| Nevada City | CA | 39.24806 | -121.002 |
| New Orleans International Airport | LA | 29.98333 | -90.25 |
| Oklahoma City Will Rogers World Airport | OK | 35.38861 | -97.6003 |
| Port Angeles Fairchild International Airport | WA | 48.12028 | -123.498 |
| Port Arthur SE Texas Regional Airport | TX | 29.95 | -94.0167 |
| Portland International Jetport | ME | 43.64222 | -70.3044 |
| Providence TF Green State Airport | RI | 41.73333 | -71.4333 |
| Punchbowl Crater 709 | HI | 21.31667 | -157.85 |
| Salt Lake City International Airport | UT | 40.76667 | -111.967 |
| Smoky Valley Carvers | NV | 38.78333 | -117.167 |
| St. Louis Lambert International Airport | MO | 38.7525 | -90.3736 |
| St. Paul Island Airport | AK | 57.15 | -170.217 |
| Tanana Calhoun Memorial Airport | AK | 65.16667 | -152.1 |
| Texas Post Office | TX | -28.8544 | 151.1681 |
| Tuscon International Airport | AZ | 32.13333 | -110.95 |
| Twin Bridges | MT | 45.55 | -112.317 |
| Valdez Weather Service Office | AK | 61.11667 | -146.267 |
| Washington Reagan National Airport | VA | 38.85 | -77.0333 |
| Wilmington New Castle CO Airport | DE | 39.66667 | -75.6 |
| Winnemucca Municipal Airport | NV | 40.9 | -117.8 |
| Wrangell Airport | AK | 56.46667 | -132.383 |
